# Mechanisms of Chromosome Number Evolution in Yeast

**DOI:** 10.1371/journal.pgen.1002190

**Published:** 2011-07-21

**Authors:** Jonathan L. Gordon, Kevin P. Byrne, Kenneth H. Wolfe

**Affiliations:** Smurfit Institute of Genetics, Trinity College Dublin, Dublin, Ireland; Washington University, United States of America

## Abstract

The whole-genome duplication (WGD) that occurred during yeast evolution changed the basal number of chromosomes from 8 to 16. However, the number of chromosomes in post-WGD species now ranges between 10 and 16, and the number in non-WGD species (*Zygosaccharomyces*, *Kluyveromyces*, *Lachancea*, and *Ashbya*) ranges between 6 and 8. To study the mechanism by which chromosome number changes, we traced the ancestry of centromeres and telomeres in each species. We observe only two mechanisms by which the number of chromosomes has decreased, as indicated by the loss of a centromere. The most frequent mechanism, seen 8 times, is telomere-to-telomere fusion between two chromosomes with the concomitant death of one centromere. The other mechanism, seen once, involves the breakage of a chromosome at its centromere, followed by the fusion of the two arms to the telomeres of two other chromosomes. The only mechanism by which chromosome number has increased in these species is WGD. Translocations and inversions have cycled telomere locations, internalizing some previously telomeric genes and creating novel telomeric locations. Comparison of centromere structures shows that the length of the CDEII region is variable between species but uniform within species. We trace the complete rearrangement history of the *Lachancea kluyveri* genome since its common ancestor with *Saccharomyces* and propose that its exceptionally low level of rearrangement is a consequence of the loss of the non-homologous end joining (NHEJ) DNA repair pathway in this species.

## Author Summary

The number of chromosomes in organisms often changes over evolutionary time. To study how the number changes, we compare several related species of yeast that share a common ancestor roughly 150 million years ago and have varying numbers of chromosomes. By inferring ancestral genome structures, we examine the changes in location of centromeres and telomeres, key elements that biologically define chromosomes. Their locations change over time by rearrangements of chromosome segments. By following these rearrangements, we trace an evolutionary path between existing centromeres and telomeres to those in the ancestral genomes, allowing us to identify the specific evolutionary events that caused changes in chromosome number. We show that, in these yeasts, chromosome number has generally decreased over time except for one notable exception: an event in an ancestor of several species where the whole genome was duplicated. Chromosome number reduction occurs by the simultaneous removal of a centromere from a chromosome and fusion of the rest of the chromosome to another that contains a working centromere. This process also results in telomere removal and the movement of genes from the ends of chromosomes to new locations in the middle of chromosomes.

## Introduction

Centromeres and telomeres are essential genetic and structural elements of eukaryotic chromosomes. To maintain the accurate transmission of the genome to the next generation, each chromosome must have exactly one centromere and two telomeres. Evolutionary changes in an organism's number of chromosomes are caused by, or result in, structural rearrangements at centromeres and telomeres. Some particular chromosome number changes have been studied in detail in other eukaryotes, such as the fusion of two chromosomes in human since the divergence from chimpanzee [1]–[2] and the insertions of whole chromosomes into other centromeres that occurred during grass evolution [3]–[4]. Here we present the first study of this kind in yeast species.

Centromeres in all eukaryotes are the site at which the kinetochore forms and is attached to spindle microtubules, which segregate sister chromosomes to opposite poles of a dividing cell during anaphase I of meiosis, and sister chromatids during mitosis and anaphase II of meiosis. They also play a role in the pairing of homologous chromosomes during meiosis [5]. Centromere malfunction can lead to aneuploidy, resulting in inviable cells or severe genetic conditions. With few exceptions, centromeres are limited to one location per chromosome, because having more than one can lead to differential attachment to opposite spindle pole bodies during cell division, causing chromosome breakage by mechanical shearing during chromosome segregation.

There are several different types of centromeres in eukaryotes [6]. Most species have ‘regional’ centromeres that are defined epigenetically and can range in size from a few kilobases, to hundreds of kilobases. These regions are often heterochromatic and contain repetitive arrays of DNA satellites. Several diverse eukaryotic species have holocentric chromosomes which are thought to have evolved independently, where the centromeric function is spread along the entire chromosome [7]. Yeasts related to *Saccharomyces cerevisiae* have a unique type of centromere, known as point centromeres [8]–[9]. These are generally less than 200 bases long and are defined by specific sequences, the CDEI, CDEII and CDEIII regions which are bound by CEN DNA-binding proteins [10]–[11]. Point centromeres are probably an evolutionary state derived from epigenetic centromeres, as more divergent fungal lineages have epigenetic centromeres that cannot be identified by sequence [12]–[13]. It has been proposed that point centromeres evolved from the partitioning elements found on selfish plasmids, which supplanted the epigenetic centromeres in the Saccharomycetaceae lineage [6]. The point centromeres in yeast are some of the fastest diverging regions in the genome [11].

Telomeres are also ubiquitous and essential in all eukaryotes. They are heterochromatic regions that serve a protective function for the chromosomes [14]–[17]. Telomeres prevent the degradation of chromosomes from their ends and stop them from being recognized as double strand breaks (DSBs). Wild type telomeres are ‘capped’ with a combination of binding proteins, chromatin structure and DNA secondary structure folding into t-loops or other higher order chromatin structures [18]–[21]. Uncapped telomeres act and are recognized as DSBs, which initiate cell cycle arrest and DSB repair pathways [19], [22]. Telomeres of *S. cerevisiae* chromosomes consist of a heterogeneous repeating sequence (basic unit TGGGTG(TG)_0–3_) that is maintained by the enzyme telomerase in an array 325±75 bp long [23]–[24]. Other species such as *Naumovozyma castellii* and *Candida glabrata* have a similar organization though the sequence and length can vary [25]. Proximal to the telomere itself is a ‘subtelomeric’ region, which in *S. cerevisiae* consists of larger repeat sequences such as the Y′ element. Further proximal again are the first genes on the chromosome, which tend to be members of subtelomere-specific repeat families such as the *DAN/TIR* and *FLO* gene families in *S. cerevisiae*.

**Figure 1:**
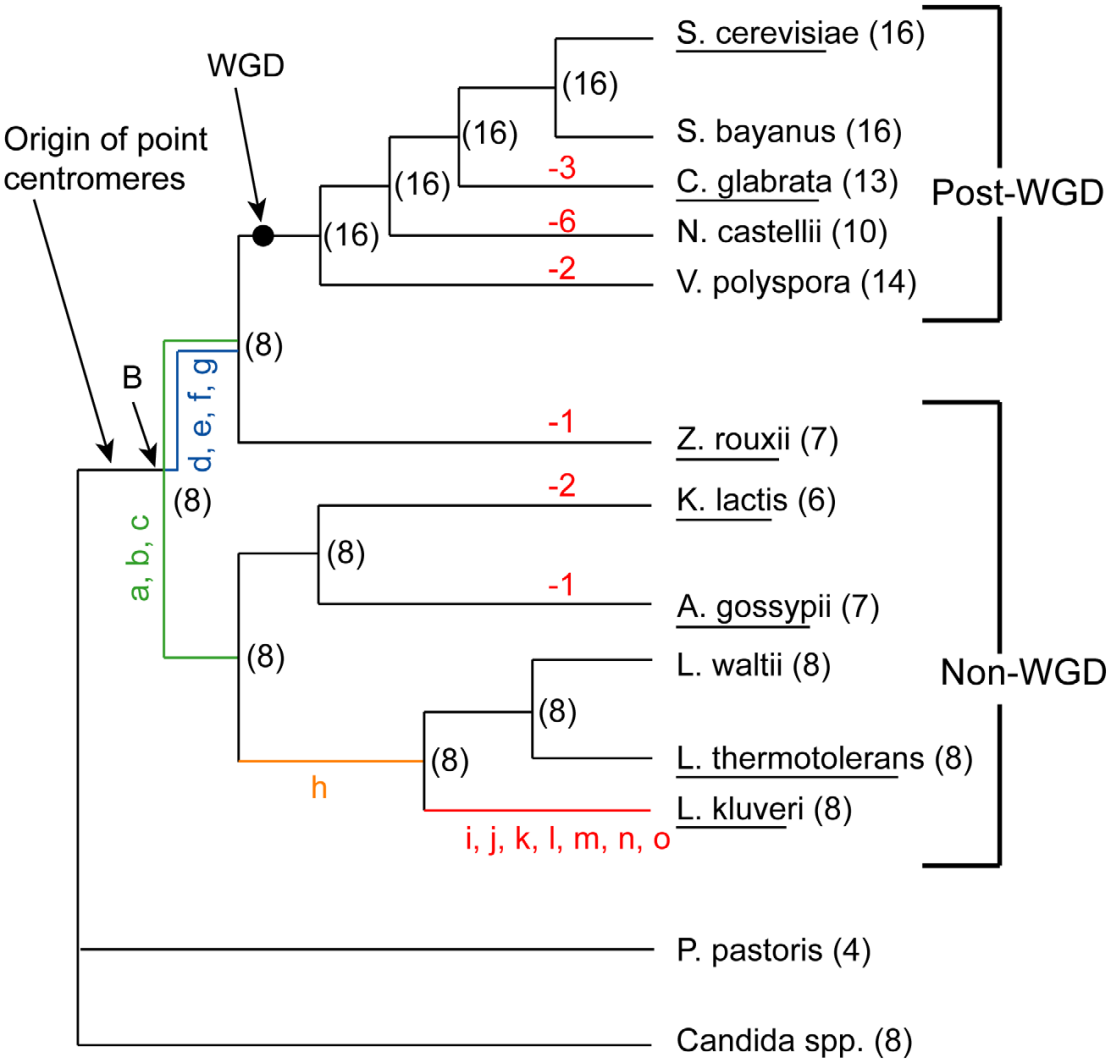
Phylogeny of the Saccharomycetaceae species used in this study. Parentheses show the numbers of chromosomes in extant species, and the inferred numbers at nodes in the tree. Negative numbers in red show chromosome number reductions. The black dot indicates the position of the WGD and the Ancestral genome sequence. Node ‘B’ is an older node that is the common ancestor of all non-WGD and post-WGD species. Lowercase letters represent specific rearrangements that differentiate *L. kluyveri* from the WGD Ancestor (black dot) as shown in Figure 2. Species whose names are underlined are those for which end-to-end complete chromosome sequences are available. The phylogeny used is that of Hedtke et al [88].

Many species from the Saccharomycetaceae family [26] have had their genomes sequenced (Figure 1) [27]–[33]. Central in this phylogeny is a whole genome duplication (WGD) event that occurred roughly 100 million years ago and gave rise to several extant paleopolyploids with reduced duplicate gene content [34]. Multiple genome sequences are available representing lineages that arose both before and after the WGD (Figure 1), referred to as non-WGD and post-WGD species, respectively [28], [33], [35].

We previously inferred the gene order and core genome structure of the ancestral species that existed immediately before the WGD [36]. This ancestral genome contained a minimum complement of roughly 4,700 genes arranged on 8 chromosomes. The WGD doubled this basal chromosome number from 8 to 16. However, many of the post-WGD species do not have exactly 16 chromosomes; *C. glabrata* for instance has only 13. Karyotype data from pulse field gel electrophoresis (PFGE) also indicates a chromosome complement that ranges between 8 and 16 chromosomes for a range of post-WGD species [37]–[38]. Similarly, some of the non-WGD species have fewer than 8 chromosomes, such as *Kluyveromyces lactis* with 6. The ancestral reconstruction has allowed us to trace the genomic rearrangements that gave rise to the genome structures of extant species. Here, we mapped the locations of the ancestral centromeres and telomeres to sites in extant species, and identified the rearrangements that caused the chromosome number to change during the evolution of these species.

## Results/Discussion

### Mapping ancestral centromere and telomere locations

We previously inferred the structure of the yeast genome as it existed immediately before the WGD occurred [36]. We refer to this genome as the ‘Ancestral genome’, and to the organism that contained it as the ‘Ancestor’. It corresponds to the point marked ‘WGD’ on the phylogenetic tree in Figure 1. The approximate locations of telomeres in this genome are already known [36]. We inferred centromere locations in the Ancestral genome by using the same parsimony approach as in [36] combined with available centromere annotations from sequenced species. The inferred Ancestral centromere locations have been included in YGOB [39]. In summary, if a centromere is present in an orthologous intergenic region in at least one non-WGD and one post-WGD species, or in paralogous ‘sister’ regions of a post-WGD species, then that centromere was inferred to have been present in the Ancestral genome (WGD node in Figure 1). We extended the inferences of centromeres and telomere locations further back along the phylogeny to the common ancestor of the non-WGD and post-WGD species (Node ‘B’ in Figure 1) to allow for inferences about the evolution of centromeres and telomeres in the genera *Kluyveromyces*, *Lachancea* and *Ashbya*.

### Lack of rearrangement in *Lachancea kluyveri*

**Figure 2:**
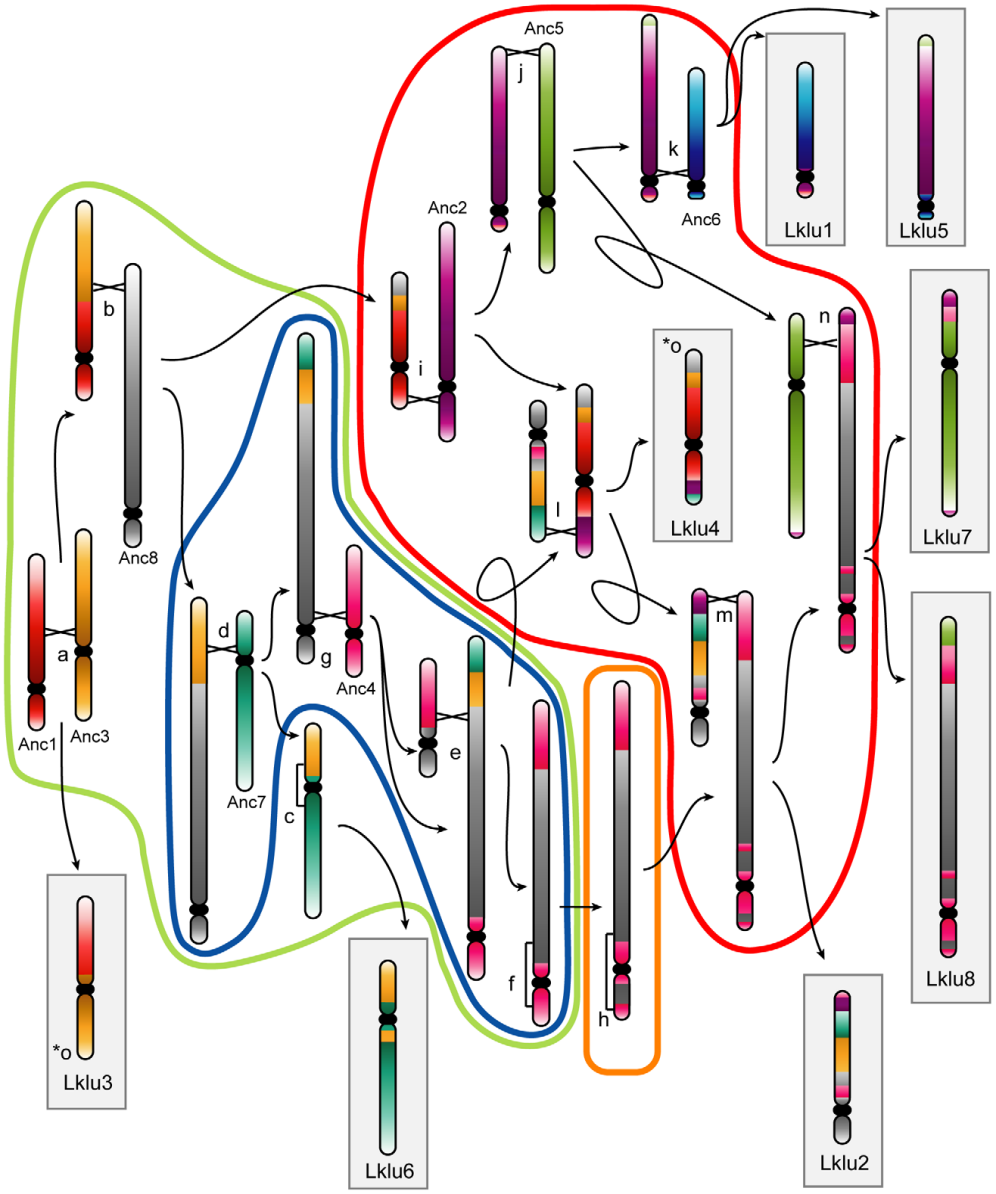
Cartoon showing the rearrangements indicated by lowercase letters in **Figure 1**. Monocolored chromosomes belong to the WGD Ancestor. Chromosomes in gray boxes are extant *L. kluyveri* chromosomes. Events encircled by a color correspond to events on branches of the same color in Figure 1. Black crossed lines between chromosomes represent points of interchromosomal translocations, and square brackets along chromosomes (events c, f and h) represent inversions. Arrows point to the products resulting from each rearrangement. The rearrangement for event o (marked with two asterisks) is not shown as it involves a reciprocal translocation located one gene from the edge of the Ancestral inference, which essentially swaps the telomeres of Anc3 and Anc8 at the ends of Lklu3 and Lklu4.

While inferring node B we found that the genome of the non-WGD species *L. kluyveri* differs from the Ancestor by only 15 rearrangements (not including inversions within synteny blocks) as shown in Figure 2 (details are given in Table S1). We then assigned these rearrangements to different branches of the tree based on their presence or absence in other non-WGD species and the outgroup *Candida* and *Pichia* clades (Figure 1). The centromere and telomere locations are nearly identical between *L. kluyveri* and the Ancestor, allowing us to infer the centromere and telomere locations in the common ancestor of the non-WGD and post-WGD species (Node ‘B’ in Figure 1).

Interestingly, by examining which Ancestral genes were not present in *L. kluyveri*, we noticed that four genes involved in non-homologous end joining (NHEJ) (*DNL4*, *POL4*, *NEJ1* and *LIF1*) are missing from the genome of *L. kluyveri* with only a degraded *DNL4* pseudogene and weak traces of an *NEJ1* pseudogene remaining in the ancestral locations. These four proteins are part of the end-processing complex which plays a role in NHEJ [40]–[42], and *DNL4*, *NEJ1* and *LIF1* are also part of the end-bridging complex [40]–[41]. NHEJ is generally limited to haploid yeast cells because the expression of *NEJ1*, a major regulator of NHEJ, is down-regulated in *MAT*a/*MAT*α diploid cells [43]–[44]. *DNL4* is required for NHEJ, and *NEJ1* regulates NHEJ, so it appears that the NHEJ pathway is missing in *L. kluyveri*. *POL4*, *NEJ1* and *DNL4* have also been shown to play roles in the alternative microhomology-mediated end joining (MMEJ) pathway, and deletions of these genes reduce the efficiency of this process several-fold [45]–[47]. We hypothesize that the loss of the NHEJ and MMEJ pathways (or a large reduction in their efficiency) in *L. kluyveri* may be linked to the low number of genomic rearrangements and lack of telomere-to-telomere fusions in this lineage. It may also be linked to the predominantly diploid lifecycle of this yeast [48], which also suggests that most DSB repair in *L. kluyveri* is through homologous recombination. Although the NHEJ machinery is not essential, to our knowledge *L. kluyveri* is the only eukaryote so far identified that lacks it. Genes for all members of the MRX and Ku complexes are still present in *L. kluyveri*, and the related species *L. thermotolerans* has a complete set of NHEJ genes.

### Mapping centromeres

The locations of centromeres were already inferred bioinformatically by the original sequencing groups for all species except *Saccharomyces bayanus*, *Vanderwaltozyma polyspora* (previously called *Kluyveromyces polysporus*) and *Naumovozyma castellii* (previously called *Saccharomyces castellii* or *Naumovia castellii*). We identified and annotated centromeres in *S. bayanus* and *V. polyspora* by extracting the intergenic regions in these species orthologous to the inferred Ancestral centromeres, and used MEME [49] to generate consensus CDEI and CDEIII profiles (full sequences of all centromeric loci are in Table S2). For *N. castellii*, Cliften et al. [50] were unable to identify any consensus centromere sequence. We too were unable to identify consensus centromere sequences at the Ancestral centromeric locations in *N. castellii* (Dataset S1). We also searched the whole *N. castellii* genome using the consensus motif for Saccharomycetaceae point centromeres derived from all identified centromeres in all species, but still could not find any candidates. Inspection of the intergenic regions corresponding to Ancestral centromeres in preliminary genome sequence data from the related species *N. dairenensis* also failed to locate any candidate point centromeres (data not shown). We hypothesize that these species may represent a novel transition of centromere structure in *Naumovozyma* which could be analogous to the earlier replacement of epigenetic centromeres by point centromeres in yeasts [6]. The system that has potentially superseded point centromeres in *Naumovozyma* will require functional characterization in the laboratory.

The correspondence between Ancestral centromere locations and current centromeres for all other extant species in the YGOB species set are shown in Table 1. All but one current centromere mapped in a straightforward manner to a corresponding Ancestral centromere with full or partially conserved syntenic gene content bordering the centromeres relative to the Ancestor. The exceptional case was *CEN9* of *C. glabrata*, which maps to Ancestral *CEN6* and has undergone a series of rearrangements with breakpoints on both sides of the centromere which have eliminated all traces of synteny at this locus (Figure S1).

**Table 1:** Mapping between Ancestral centromeres and centromeres in extant species.

Ancestral	*S. cer.*		*S. bay.*		*C. gla.*		*V. pol.*		*Z. rou.*	*K. lac.*	*A. gos.*	*L. klu.*	*L. the.*	*L. wal.*
**CEN1**	3**+**	14**+**	3	14	2**r**	**X**	s1050	**Xr**	5	1**r**	1	4*	8	s0
**CEN2**	8**−**	11**−**	8	11	11**i**	**X**	s1056*	s1018	**X**	2	2	1*	4*	s26*
**CEN3**	2**+**	4**+**	2	4	3*	13	s1036	s1045	1	**X**	5	3	7*	s27*
**CEN4**	1**+**	7**−**	1	7	1**i**	7**r**	s1062	**X**	7	3*	6	8*	5*	s47*
**CEN5**	10**−**	12**−**	10	12	4	**X**	s534	s2002	2	6**r**	**Xr**	7*	2*	s33*
**CEN6**	13**+**	15**−**	13	15	5	9**?r**	s1032	s1037	3	**X**	3	5	3	s56
**CEN7**	5**+**	9**+**	5	9	8**r**	10	s499	s312	4	4*	4	6	6	s55
**CEN8**	6**+**	16**+**	6	16	6	12	s354	s1058	6	5*	7	2	1	s23
Total	16		16		13		14		7	6	7	8	8	8

The first column lists the Ancestral centromeres, and the numbers in the subsequent columns lists chromosome numbers (or scaffold numbers for unfinished genomes) where the orthologous centromeres are found in the other species. Post-WGD species have up to two centromeres for each ancestral centromere. The final row lists the total number of chromosomes in each species.

**X**, Centromere lost.

**+/−**, Sense/anti-sense strand in *S. cerevisiae*.

*, Orientation change.

**?**, Possible orientation change (see text).

**r**, Reciprocal translocation at centromere.

**i**, inversion at centromere.

### Mapping telomeres

We traced the evolution of telomere locations in all the species for which completely finished genome sequences are available, but not for those whose genomes consist of numerous scaffolds, due to the uncertainty in identifying real telomeric regions in scaffold data (Table 2). In most of the genomes, mapping the current telomeres to Ancestral locations is relatively trivial as there is a direct correspondence without genome rearrangements at those locations (Table 2). However in *C. glabrata*, *A. gossypii* and *K. lactis* several telomeres mapped to Ancestral locations through a complex set of rearrangements including breakpoint reuse. The genomes of these species are also the most rearranged of those examined. By contrast, members of the *Lachancea* clade have had relatively few genomic rearrangements on the evolutionary path between them and the Ancestor. The mapping of telomeres to Ancestral telomeres is more tentative than for the centromeric mapping, due to the inherently unstable nature of telomeres, and the possibility of movement of the telomeric boundaries. For example, if we had genome sequences from more species, it might become possible to extend the Ancestral genome inference further towards the telomeres and so reveal rearrangements that are presently inaccessible that may alter the mapping. The current telomere assignments represent the most parsimonious mappings given the data that is currently available.

**Table 2:** Mapping between Ancestral telomeres and telomeres in extant species with finished genome sequences.

Anc.	End	*S. cer.*		*C. gla.*		*Z. rou.*	*K. lac.*	*A. gos.*	*L. klu.*	*L. the.*
**1**	**L**	3-L	**X** (4-R)**i**	2-L	7-R	6-R	3-L	6-R	3-L	6-L
	**R**	**X** (2-L)*	11-L	6-L	11-L	2-L	Anc8L*†‡	5-R	1-R	2-L
**2**	**L**	**X** (10-L)*	4-L	**X** (12-L)*	3-R	**X** (3-R)*†	4-R	2-L	7-L	8-L
	**R**	**X** (2-R)**i**	9-R	9-L	**X** (10-L)*†	4-L	**X** (1-L)*	**X Anc4-R** (1-R, 6-L)**‡**	7-R	3-L
**3**	**L**	13-R	14-L	13-R	Anc5-L*†‡	**X** (1-R)*†	3-R	7-R	4-L	5-R
	**R**	7-L	6-R	**X** (8-R)*†	1-R	5-R	Anc6-R*†‡	**X** (1-L)*†	6-L	7-L
**4**	**L**	12-L	8-L	**Anc6-R†‡**	**X** (10-R)*†	**X** (7-R)*†	5-L	**X** (4-L)*†	8-R	8-R
	**R**	10-R	8-R	13-L	6-R	7-L	**X** (1-R)*†	**X Anc2-R** (1-R, 6-L)**‡**	2-R	**X** (4-R)**i†**
**5**	**L**	15-L	7-R	Anc3-L*†‡	7-L	Anc8-L*†‡	**X** (6-R)*†	2-R*	**X** (8-L)**i**	**X** (2-R)
	**R**	9-L	11-R	9-R	Anc8-R*†‡	2-R	2-L*†	7-L	5-R	5-L
**6**	**L**	**X** (16-L)**i**	**X** (5-L)*	**X** (1-L)*†	**X** (12-R)*	6-L	4-L	**X Anc8-R** ^•^ **†**	5-L	3-R
	**R**	14-R	3-R	**Anc4-L†‡**	5-L	4-R	Anc3-R*†‡	**X** (3-R)*†	1-L	1-R
**7**	**L**	1-L	15-R	4-L	4-R	3-L	**X** (5-R)*†	4-R	4-R	4-L
	**R**	16-R	12-R	**X** (5-R)*†	8-L	5-L	**X** (6-L)*†	3-L	6-R	1-L
**8**	**L**	**X** (5-R)*†	13-L	**X** (2-R)*†	11-R	Anc5-L*†‡	Anc1R*†‡	**X** (5-L)*i	2-L	7-R
	**R**	**X** (1-R)*	6-L	Anc5-R*†‡	3-L	1-L	2-R*†	**X Anc6-L** ^•^	3-R	6-R

The first column lists Ancestral chromosome numbers, and the second column lists the chromosome ends. For each Ancestral chromosome end, the corresponding orthologous chromosome end for each species examined with a finished genome sequence is given in the same column. Post-WGD species have two chromosome ends that correspond to each Ancestral chromosome end. Many of the corresponding chromosome ends have undergone rearrangements including fusions to other chromosomal locations which have led to the death of the Ancestral location, and in some cases the birth of a new telomere elsewhere in the genome.

**X,** Loss of Ancestral telomere (newly created telomere in parentheses).

**AncX-L/R,** Fusion to another Ancestral telomere.

*, Rearrangement by translocation.

**†**, Internalization of genes.

**‡**, Chromosome fusion.

**i**, Inversion.

**•**, *A. gossypii* unique loss of centromere and telomeric fusion of chromosome arms.

### Centromere losses

**Figure 3:**
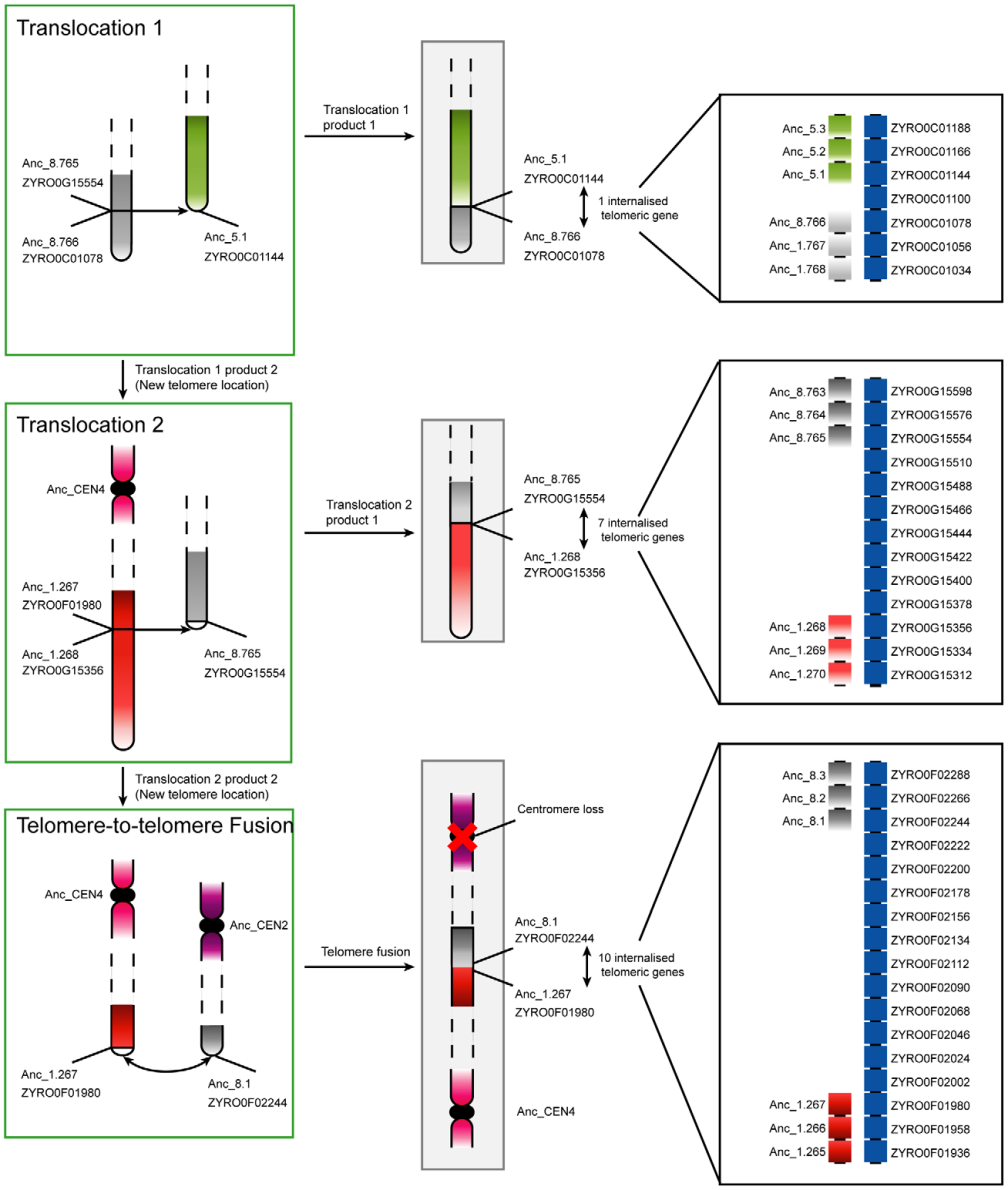
Progression of rearrangements and chromosome fusions leading to the loss of a centromere in *Z. rouxii*. Two non-reciprocal telomeric translocations and a telomere-to-telomere fusion gave rise to the extant chromosome structures in *Z. rouxii*. Chromosomes in green boxes are those that underwent rearrangements, while those in gray boxes are finished translocation products (*i.e.*, extant regions in *Z. rouxii*). The edges of the breakpoints are labelled with both the Ancestral and current *Z. rouxii* gene names. In the bottom step, the loss of a centromere occured contemporaneously with the two chromosomes fusing at their telomeres. All three rearrangements led to the internalisation of previously telomeric genes. The panels on the right show details of the gene orders and internalized telomeric genes at the junctions.

We identified nine losses of a centromere, corresponding to nine decreases of chromosome number. Three of these occurred in *C. glabrata*, two each in *V. polyspora* and *K. lactis*, and one each in *Z. rouxii* and *A. gossypii* (Figure 1). The major mechanism of centromere loss was associated with the telomere-to-telomere fusion of two chromosomes with the loss of one of the centromeres. This mechanism is illustrated by the chromosome fusion and single centromere loss that occurred in *Z. rouxii*, whose details are shown in Figure 3. In this example, the process also resulted in the internalization of many genes that were previously located near telomeres. All but perhaps one of the nine centromere losses occurred in this fashion, resulting in the loss of at least 14 of the 112 telomere locations examined. The removal of centromeres appears to have been quite specific, generally leaving adjacent genes intact. In some cases a centromere and some adjacent genes are missing, but all these cases occur in post-WGD species where gene deletion is relatively common due to the redundancy created by the WGD. None of the centromere losses in non-WGD species is accompanied by loss of centromere-adjacent genes.

The majority of centromere losses in yeast appear to have involved the fusion of whole chromosomes. In these cases, two possible scenarios exist that differ only in the order of events. The first scenario is the initial fusion of the chromosomes at telomeric locations, with subsequent loss of one of the two centromeres. In this case selection would likely act to suppress one of the two centromeres to avoid problems during cell division. The second scenario is that the centromere of a chromosome is first lost or disabled, with the chromosome subsequently being rescued from cellular loss by fusion to another chromosome with a functional centromere. Under the latter scenario, selection acts to maintain the genes contained on the chromosome without a centromere, because cells missing a whole chromosome will certainly be inviable. Chromosome fusions have been generated experimentally in *S. cerevisiae* by the inactivation of a centromere [51]. Interestingly, if the centromere is reactivated, it often leads to fission of the resulting chromosome at or near the fusion site to reconstitute the parental karyotype [51], indicating that the fusion point may be a fragile site. This fragility might explain the reuse of fission/fusion breakpoints like those shared between Translocations 1, 2 and 3 in Figure 3.

**Figure 4:**
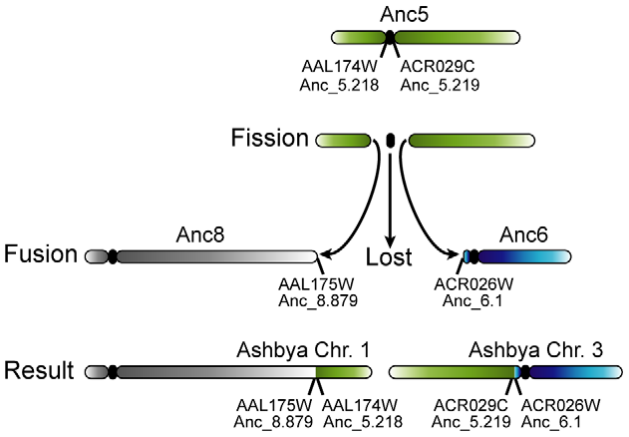
Loss of a centromere in *A. gossypii* by the breakage of a chromosome at its centromere. The green chromosome at the top represents chromosome 5 at Node ‘B’ of the tree (Figure 1), which is identical to chromosome 5 of the WGD Ancestor (see Figure 2). After *A. gossypii* diverged from *K. lactis*, this chromosome broke in the intergenic region containing its centromere. To avoid losing large numbers of genes during cell division, both arms of the split chromosome fused their broken edges to the telomeres of Ancestral chromosomes 6 and 8, which gave rise to the organisation on the extant *A. gossypii* chromosomes 1 and 3. The timing of loss of the centromere is unclear: it may have happened as a part of the rearrangement, or the centromere may have been carried on one of the chromosome arms and lost after fusion to the telomere of another centromere-containing chromosome. The mechanism of the fission event is also ambiguous: it may have occurred by the chromosome actually breaking into two, or by two separate translocations to other chromosome ends that separated the centromere from its neighboring genes.

The unique case observed in *A. gossypii* appears to have occurred by the breakage of a chromosome in the intergenic region that contained Ancestral centromere Anc_CEN5 (Figure 4). The resulting two chromosome arms then fused to two other chromosomes, joining the previously centromere-proximal sequences to the telomeres of the other chromosomes. The exact nature of this fission and fusion is not known, and we cannot tell the difference between chromosome breakage and religation to new locations, or translocation events. It is also not possible to infer whether the centromere was destroyed in the fission event, or whether it was still intact at the end of one of the arms that subsequently fused to another telomere and was lost later due to the constraint of having one centromere per chromosome.

We observed no cases of *de novo* centromere gain. Apparently, the only mechanism by which chromosome number has increased during the evolution of Saccharomycetaceae is WGD (Figure 1). This discovery is quite surprising, because the spontaneous formation of aneuploids with duplications of single centromeres or chromosomes has frequently been reported, both in *S. cerevisiae* [52]–[53] and *C. glabrata* [54]. Interestingly, from the sequenced genomes only species in the genus *Saccharomyces* have retained all 16 centromeres from the WGD, while the other sequenced post-WGD species (*V. polyspora*, *N. castellii* and *C. glabrata*) all have a reduced chromosome complement that arose independently in their respective lineages (Figure 1). Previous PFGE karyotype analyses indicated that some strains of *Kazachstania exigua* may also have a chromosome complement of 16 [37]–[38], the most likely explanation of which is that this species has also retained all of its centromeres since the WGD.

### Consensus centromere sequences

We compiled and compared the CDE consensus sequences for all sequenced yeasts with point centromeres (Figure S3). All the centromeres of *S. cerevisiae* have been characterized functionally [8]–[9], and a few have been cloned from other yeasts: *S. bayanus* [55]–[56], *C. glabrata* [57], *Z. rouxii* [58] and *K. lactis* [59]. The genome sequencing groups made bioinformatic predictions about centromere locations for most of the other chromosomes and species, based on matches to the CDEI–III consensus sequences [27]–[28], [30], [33]. We used these in our analysis, though we revised the coordinates of two *L. waltii* centromeres (Table S4). We identified CDE regions for centromeres in *S. bayanus* (Table S5) and *V. polyspora* (Table S6), finding 16 and 14 centromeres respectively. Although the genome sequence of *V. polyspora* is incomplete [32], there is complete intergenic sequence spanning both of the lost centromeres meaning we are confident of their absence. Our count of 14 centromeres is one more than the previous estimate of chromosome number in this species [60].

**Figure 5:**
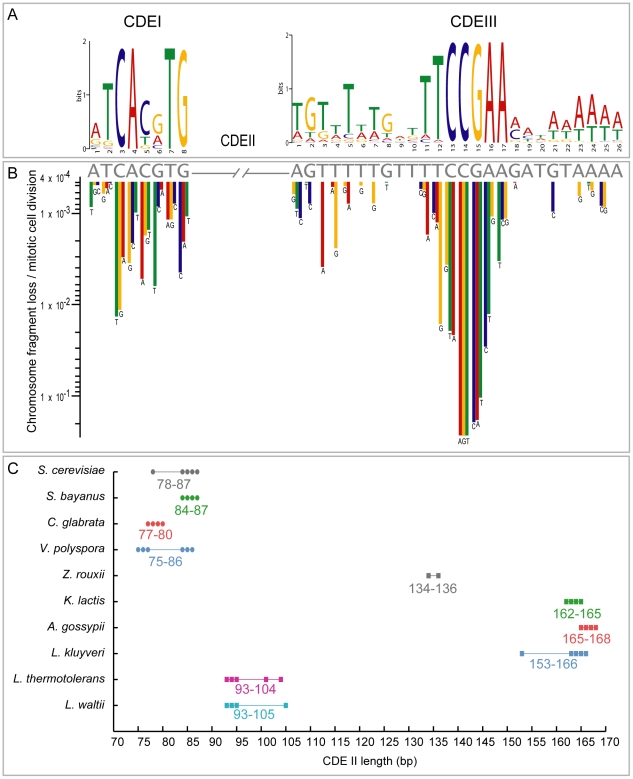
CDE conservation in the Saccharomycetaceae. (A) Sequence logo showing base frequencies at each position in all annotated CDEI and CDEIII regions from 10 species. (B) Rate of chromosome loss per mitotic cell division caused by mutagenesis of individual residues in CDEI and CDEIII sequences (gray letters) of *S. cerevisiae CEN6* (redrawn from [61]). Sites conserved in the logo tend to have the largest effects on chromosome loss when mutated. (C) Variation of CDEII lengths in species with identifiable point centromeres. The number of points is fewer than the number of chromosomes in each species because some chomosomes have identical CDEII length.

With over a hundred yeast centromeres in our dataset we searched for features common to all point centromeres (Figure 5). For consistency with *S. cerevisiae*, in this analysis we delineated the boundaries of CDEI, CDEII and CDEIII regions in the same way across all genomes disregarding small differences in the boundary choices made by different sequencing groups. The CDEI regions have an 8 bp consensus motif with four invariant sites (NNCAVBTG). The CDEIII regions have an invariant 5 bp motif (CCGAA) and the whole CDEIII consensus is 26 bp. Within a given species there are often further invariant sites in their CDEI or CDEIII regions, for example G at positions 2 and 8 in *S. cerevisiae* CDEIII. The intervening CDEII regions are always highly AT-rich (76–98%). The length of CDEII varies twofold among species, but there is remarkably little CDEII length variation within each species, and a clear correlation of CDEII lengths among related species (Figure 5C).

Hegemann and Fleig [61] compiled and summarized mutagenesis studies on *S. cerevisiae CEN6* [62]–[64], measuring the frequency of chromosome fragment loss resulting from point mutations at many sites in *CEN6*. There is a strikingly strong correlation between their results and the evolutionary conservation of individual sites in CDEI and CDEIII (Figure 5A, 5B). None of the 13 nucleotide changes with the most severe phenotypes (chromosome fragment loss rates >10^−2^ per mitotic cell division) at *CEN6* occurs as a natural variant in the 102 centromeres we compiled. Thus the evolutionary conservation of these regions over hundreds of millions of years correlates well with the highest impact point mutations from the mutational data. Due to these constraints, we suggest that the *de novo* formation of a point centromere in these yeast species is much less likely than the *de novo* creation of regional centromeres in other species such as *Candida albicans* [65] because heritable epigenetic changes can occur on a much smaller timescale than sequence-based evolution.

### Rearrangements at centromeres

Reciprocal translocation and inversion breakpoints were observed adjacent to centromeres in *C. glabrata*, *V. polyspora*, *A. gossypii* and *K. lactis*, as were orientation changes of the centromeres (Table 1). *V. polyspora* and *A. gossypii* each show only one such event, and in both cases the rearrangement breakpoints coincide with the site of a centromere loss in these species. *K. lactis* has three rearrangement breakpoints adjacent to centromeres, and *C. glabrata* has six, none of which coincide with centromere loses in either species. Interestingly, the breakpoints adjacent to the three centromeres in *K. lactis* are all part of one rearrangement cycle (Figure S2), indicating that there have been reciprocal translocations between intergenic locations containing centromeres.

### Telomere cycling and internalization of telomeric genes

Translocations causing a terminal segment of one chromosome to be transferred and joined to another chromosome were observed in *Z. rouxii* (Figure 3), *S. cerevisiae*, *C. glabrata*, *K. lactis* and *A. gossypii*. As well as physically moving an existing telomere to a new chromosome, this type of rearrangement results in some previously subtelomeric DNA becoming internal to chromosomes where the fusion occurred (Figure 3). These events can be inferred at the level of synteny blocks, but they probably occurred millions of years ago and there is currently no telomere-like DNA sequence at the rearrangement points. Conversely, previously internal regions on the chromosomes located at the breakpoints of telomeric translocations become novel telomere sites (*e.g.*, gene *ZYRO0G15554* after Translocation 1 in Figure 3, before it became the join-site of another telomeric translocation). Analogous birth and death of telomere locations can occur by inversions and are found in *S. cerevisiae*, *A. gossypii*, *Z. rouxii* and *K. thermotolerans* (Table 2). Telomeric translocations and inversions have resulted in the turnover of more than a quarter (33/112) of telomere locations relative to the ancestor. As well as inversions and translocations, the death of telomere locations can be caused by telomere-to-telomere fusions. The gain of novel telomere sites is presumably by telomere capture, a process that has been observed in cells that survive the absence of telomerase or defective telomere capping. Novel telomeres can also be generated at the site of a DSB by telomerase, a process that is enhanced by G-rich telomeric seed sequences lying close to the DSB [66]–[69].

Internal chromosomal positions differ from subtelomeric locations in terms of their chromatin configurations, which in turn affect the expression of nearby genes [70]–[72]. In general, subtelomeric regions tend to have higher nucleosome occupancy and silencing protein association, both of which generally reduce gene expression [70]–[72]. Subtelomeric genes are likely to be under less evolutionary constraint than genes in internal locations, are less essential and have higher variance in their expression profiles [73]. The rate of sequence evolution is negatively correlated with expression and essentiality, but positively correlated with the variance of gene expression [74]–[77]. Thus relocating a gene from telomeric to internal regions is likely to increase the evolutionary constraints on its sequence. Conversely, evolution may proceed at a faster pace at telomeres due to more relaxed selective constraints. If this higher evolutionary rate leads to an advantageous allele at a telomere, we hypothesize that it may be beneficial to relocate the gene to somewhere else in the genome where selection will maintain the advantageous allele under higher constraint. This could potentially constitute an ongoing cycle over evolutionary time, where the telomeres act as the cooking pots of evolution [78], with successful innovations moving to more stable regions.

Rearrangements that internalize genes appear to be more common in genomes that have high rates of genome rearrangement. In *S. cerevisiae*, which is the least rearranged post-WGD species [36], only two genes (*GAL2* and *SRL2*, which are in the same breakpoint location) were internalized by rearrangement from a telomere (Table S3). In *C. glabrata*, arguably the most rearranged post-WGD species [36], there are at least 17 internalized genes in 8 locations (Table S3) even though the telomeres of *C. glabrata* contain many fewer annotated genes than those of *S. cerevisiae*. Non-WGD genomes that have high levels of rearrangement such as *K. lactis* and *A. gossypii* [36] contain high numbers of these genes (at least 48 genes in 19 locations and 15 genes at 8 locations respectively) (Table S3). In *Z. rouxii*, which is intermediate in terms of rearrangement, there are at least 27 genes at 7 locations, while in the rearrangement poor *L. thermotolerans*, there are 6 genes at a single location. There are no internalized genes in the *L. kluyveri*, the least rearranged non-WGD species. These numbers also somewhat reflect the overall numbers of subtelomeric genes annotated in these species.

Large scale genomic rearrangements like the fusions of telomeres to other telomeres or internal chromosomal sections inferred in this work are generally considered to be detrimental to cells although they are not necessarily so. Many cancers involve similar types of rearrangements, and there are several pathways and mechanisms in place in cells to prevent and repair them, including proteins involved in telomere structure and maintenance, cell cycle arrest signalling, homologous recombination (HR) and NHEJ repair pathways [19], [22], [69], [79]–[81]. Interestingly, many of the components of the HR and NHEJ machinery such as the MRX complex, Yku70/80 proteins and Rad17/Mec3/Ddc1 complex also play roles in telomere structure and stability and are associated to telomeres [19], [22], [79]–[81]. Experimental deletions of genes involved in these pathways as well as those involved in telomeric structure have helped to tease apart their functions at telomeres, and many of the deletions result in chromosomal rearrangements such as telomere-to-telomere fusions and non-reciprocal translocations, similar to those inferred in our work [19], [22], [80]–[82]. The gross chromosomal rearrangements observed in these mutants generally manifest through a NHEJ-like mechanism requiring Dnl4 (Lig4), an NHEJ ligase [79]–[81].

Spontaneous rearrangements involving telomere fusions to other telomeres or DSBs occur in wild type *S. cerevisiae* cells at a rate of 1–6×10^−7^ events per genome per cell division [80], but have only been fixed a few times throughout Saccharomycetaceae evolution. Together with evidence that *S. cerevisiae* is capable of rescuing cells from DSBs by telomere capture at the edge of the DSB from the centromere-containing part of the chromosome [66], [68], [83], it appears that telomeric rearrangements such as telomere-to-telomere fusions and non-reciprocal translocations likely represent rare errors in the systems that protect and cap telomeres or repair DSBs that have been fixed over evolutionary time. It is only possible to speculate about the exact causes of the rearrangements, how they became fixed in populations, and whether they were selectively advantageous, neutral or disadvantageous. The observed rearrangements are in the order of millions of years old, and are thus unlikely to contain any sequence information that could provide empirical evidence about their mechanism of formation.

We suggest that the rearrangements probably occurred in haploid cells, as in a diploid it would be expected that DSBs would be repaired via homologous recombination using the homologous chromosome as templates. In the Saccharomycetaceae where mating-type switching occurs [28], [84], rearrangements in haploids would also avoid mating incompatibilities that could arise in a diploid due to meiotic segregation difficulties [85]. A haploid cell could divide, change mating type and then mate with the daughter cell, thus avoiding potential chromosome pairing problems and aneuploidy.

### Perspectives

Among the species studied here (the family *Saccharomycetaceae*) [26], we find that chromosome number has evolved by two very different mechanisms. The only mechanism of increase was polyploidization. We suggest that the lack of any other new centromere formation is a consequence of the sequence-defined nature of point centromeres, but it is unclear why the formation of a new centromere by small-scale DNA duplication of an existing centromere, as seen in *C. glabrata* drug resistance isolates [54], is not seen during evolution. The mechanism of decrease in chromosome number was by rearrangements involving telomeres, primarily telomere-to-telomere fusions with the loss of a centromere belonging to one of the fused chromosomes. The temporal sequence of the chromosome fusion and centromere loss is ambiguous. Telomeric rearrangements have also frequently moved genes from subtelomeric locations to internal genomic locations. These movements have the potential to change the selective constraints on the genes and could be evolutionarily adaptive.

## Materials and Methods

### Mapping centromeres and telomeres to the Ancestor

The Ancestral centromere locations were generally trivial to find because numerous comparisons among extant non-WGD and post-WGD species can be made, most centromere locations are in syntenic regions among species, and most rearrangements that might obscure these relationships are species specific. Ancestral centromere loci were added to YGOB following the same parsimony rules as in [36], by using species for which centromere annotations have already been made. These Ancestral centromere locations were then used to guide the search for unannotated centromeres in orthologous intergenic regions by searching for CDEI and CDEIII sequence motifs using MEME [49].

To map the rearrangements that had occurred at a centromere in any particular species, we examined the breakpoints between synteny blocks in that species relative to the Ancestor and tried to locate the reciprocal breakpoint elsewhere in the genome. In some cases, a reciprocal breakpoint did not exist; these cases represent breakpoint reuse [36]. They can be solved by following one edge of the breakpoint (A|B) locating the reciprocal edge at another location (B′|C), then finding the breakpoint partner's reciprocal edge (C′|D) and iterating this process until reaching the original breakpoint's other edge (D′|A′). This process identifies a cycle of breakpoint edges that eventually leads back to the adjacent edge of the centromeric breakpoint.

Telomeric locations were mapped between the Ancestor and extant species in a similar way, except the extant telomere positions were defined as the regions at the ends of chromosomes where it is no longer possible to define Ancestral genes based on synteny across species, *i.e.* the regions in extant species that lie beyond the edges of the Ancestral chromosome reconstruction. As telomeres have a very high rate of rearrangement, we regard telomeres as locations rather than as any particular genes. Thus the telomere locations of a chromosome were defined as the locations beside the leftmost and rightmost genes on that chromosome that have orthologs in the Ancestral genome. We only analyzed the evolution of telomere locations in species whose genomes are completely sequenced, because for incompletely sequenced species we cannot be sure that there is a telomere at the end of each scaffold.

To trace the evolution of centromere and telomere positional evolution in the non-WGD species, which are not direct descendants of the Ancestor (Figure 1), we mapped the translocational rearrangements between the Ancestor and the non-WGD species *L. kluyveri* onto the phylogeny by comparing their presence and absence in other extant species in the Saccharomycetaceae and outgroups (*Pichia pastoris* [86] and the *Candida* clade of species [87]).

### Absence of NHEJ genes in *L. kluyveri*

The four genes involved in NHEJ that are missing from *L. kluyveri* were identified by compiling a list of genes in the YGOB database that are present in the Ancestral genome but not in the *L. kluyveri* genome. We noticed that four genes in the list had a role in NHEJ. We then examined the *L. kluyveri* intergenic locations where these genes would be expected to reside, to make sure that they were not present but unannotated. No potentially coding ORFs were found in these regions, but pseudogene relics of *DNL4* and *NEJ1* were identified. Finally, protein sequences from the four genes from the closely related *L. thermotolerans* were used as TBLASTN queries against the *L. kluyveri* chromosome sequences to make sure they were not present elsewhere in the genome.

## Supporting Information

Dataset S1 Intergenic regions in *N. castellii* orthologous to Ancestral CEN loci. Text file containing FASTA sequences from *N. castellii* candidate CEN regions based on Ancestral CEN locations. Only 10 of the possible 16 candidate intergenic regions could be unambiguously identified due to multiple rearrangements in 6 of the candidate regions.
(TXT)

Figure S1 Rearrangement path between Ancestral *CEN6* and *C. glabrata CEN9*. The blue chromosome at the top left represents the chromosomal regions adjacent to the centromere (black dot) on Ancestral chromosome 6. Each block consisting of a single color gradient represents an Ancestral chromosome region, prior to rearrangement. Genes adjacent to breakpoints are labelled for both the Ancestor and *C. glabrata*. Each reciprocal translocation is represented by a red cross extending between two chromosome segments and results in two translocation products (marked by arrows). Rearrangement products outlined with a green box represent final arrangements in *C. glabrata*, while those boxed in red are intermediate products that undergo further rearrengements with other Ancestral-type regions. There are nine reciprocal translocations in this rearrangement pathway, which removes all traces of Ancestral synteny from *C. glabrata CEN9*, and involves the reuse of eight breakpoints. The ordering of events in this cartoon is only one possible permutation of many, as there are many possible orders of events depending on which of the two breakpoint edges from the unfinished product is chosen to undergo rearrangement at each step.
(TIF)

Figure S2 Rearrangement cycle with breakpoint reuse at three centromeric locations in *K. lactis*. The cycle involves four reciprocal translocation events, three of which occur at Ancestral centromere positions. Each Ancestral centromere-adjacent region is represented by a color gradient block and orange Ancestral gene names. Centromeres are represented by black circles. Reciprocal translocation events are represented by colored lines joining the gradient blocks. Three of the reciprocal translocations produce one ‘finished’ product (indicated by a green arrow, outlined by a green box and with blue *K. lactis* gene names), which is a current adjacency in the *K. lactis* genome, and one ‘unfinished’ product (indicated by a red arrow), which will undergo further rearrangement. The final reciprocal translocation produces two ‘finished’ products.
(TIF)

Figure S3 Consensus MEME logos for CDEI and CDEIII motifs in the species examined. Species are split into post-WGD and non-WGD. Length range and %AT range is shown for the CDEII region.
(TIF)

Table S1 Breakpoint edges for the 15 rearrangements between the Ancestor and *L. kluyveri*. The rearrangements between the Ancestor and *L. kluyveri* are labelled (a-o), and correspond to the events in Figure 1. Each edge gene at the Ancestral breakpoint location is shown with the corresponding ortholog in *L. kluyveri*. The rearrangement products which are adjacent in the extant *L. kluyveri* genome unless further intra-synteny block inversion occurred (signified with an asterisk) are in the two rightmost columns.
(XLS)

Table S2 CEN sequences. The chromosome/scaffold, coordinates, length and sequence for each extant centromere in the species examined corresponding to each Ancestral centromere are shown.
(XLS)

Table S3 Previously subtelomeric genes internalised into core chromosome locations in each species. Genes internal to chromosomes that were previously in subtelomeric locations in the species *S. cerevisiae*, *C. glabrata*, *Z. rouxii*, *K. lactis*, *A. gossypii* and *L. thermotolerans*.
(XLS)

Table S4 CDE Consensus sequences in *L. waltii*. Sequences and length details of the CDEI, CDEII and CDEIII sequences and the overall CEN coordinates in *L. waltii*.
(XLS)

Table S5 CDE Consensus sequences in *S. bayanus*. Sequences and length details of the CDEI, CDEII and CDEIII sequences and the overall CEN coordinates in *S. bayanus*.
(XLS)

Table S6 CDE Consensus sequences in *V. polyspora*. Sequences, length details of the CDEI, CDEII and CDEIII sequences, scaffold numbers, coordinates and GenBank accession numbers for the centromeres of *V. polyspora*.
(XLS)
