# Supplementary figures and images for: Mechanisms of Chromosome Number Evolution in Yeast
Source: PLoS Genet. 2011 Jul 21;7(7):e1002190. doi: 10.1371/journal.pgen.1002190 (PMC3141009; doi:10.1371/journal.pgen.1002190)

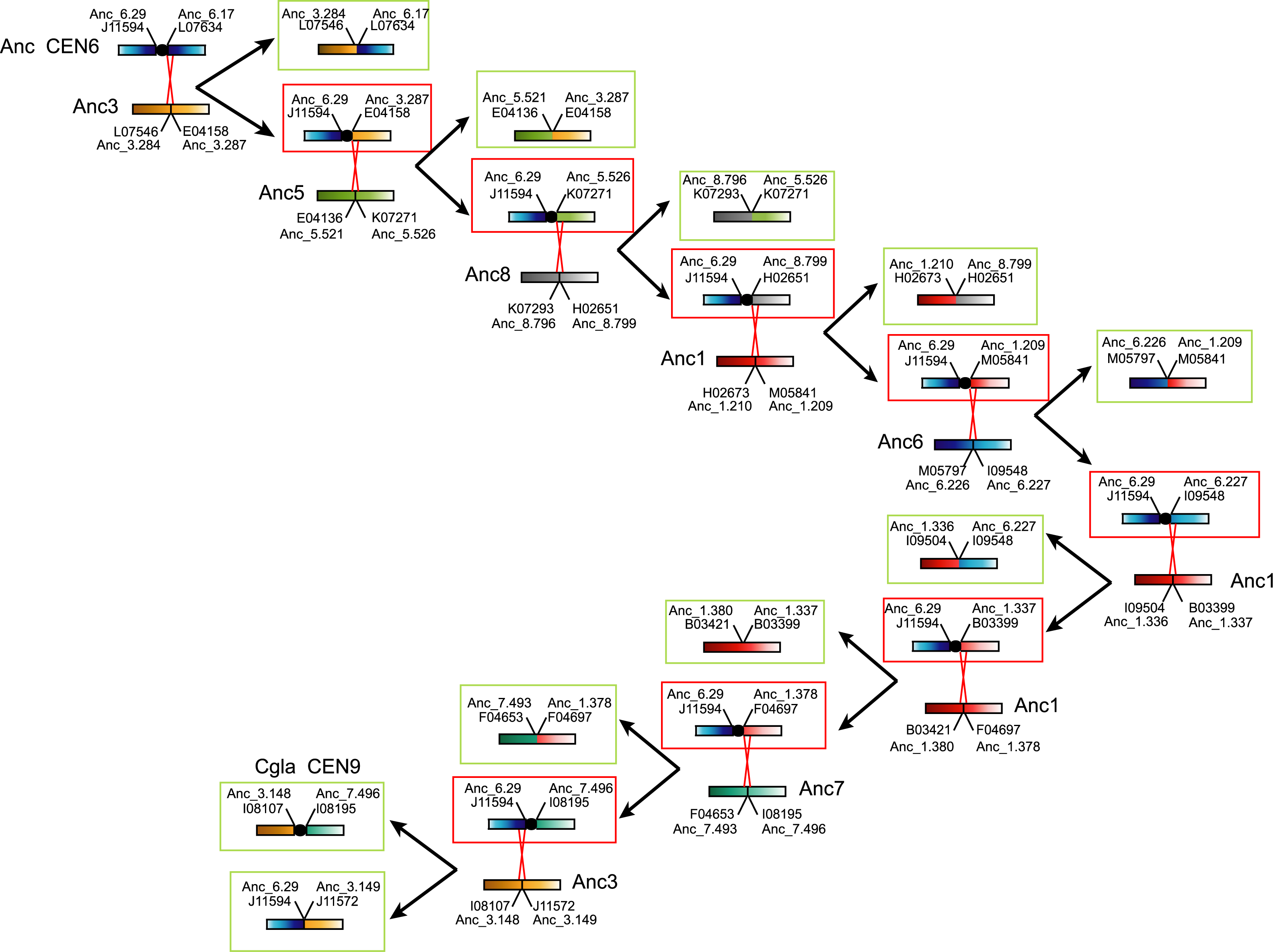

Supplement: Figure S1 — Rearrangement path between Ancestral CEN6 and C. glabrata CEN9. The blue chromosome at the top left represents the chromosomal regions adjacent to the centromere (black dot) on Ancestral chromosome 6. Each block consisting of a single color gradient represents an Ancestral chromosome region, prior to rearrangement. Genes adjacent to breakpoints are labelled for both the Ancestor and C. glabrata. Each reciprocal translocation is represented by a red cross extending between two chromosome segments and results in two translocation products (marked by arrows). Rearrangement products outlined with a green box represent final arrangements in C. glabrata, while those boxed in red are intermediate products that undergo further rearrengements with other Ancestral-type regions. There are nine reciprocal translocations in this rearrangement pathway, which removes all traces of Ancestral synteny from C. glabrata CEN9, and involves the reuse of eight breakpoints. The ordering of events in this cartoon is only one possible permutation of many, as there are many possible orders of events depending on which of the two breakpoint edges from the unfinished product is chosen to undergo rearrangement at each step. (TIF) [file pgen.1002190.s002.tif]

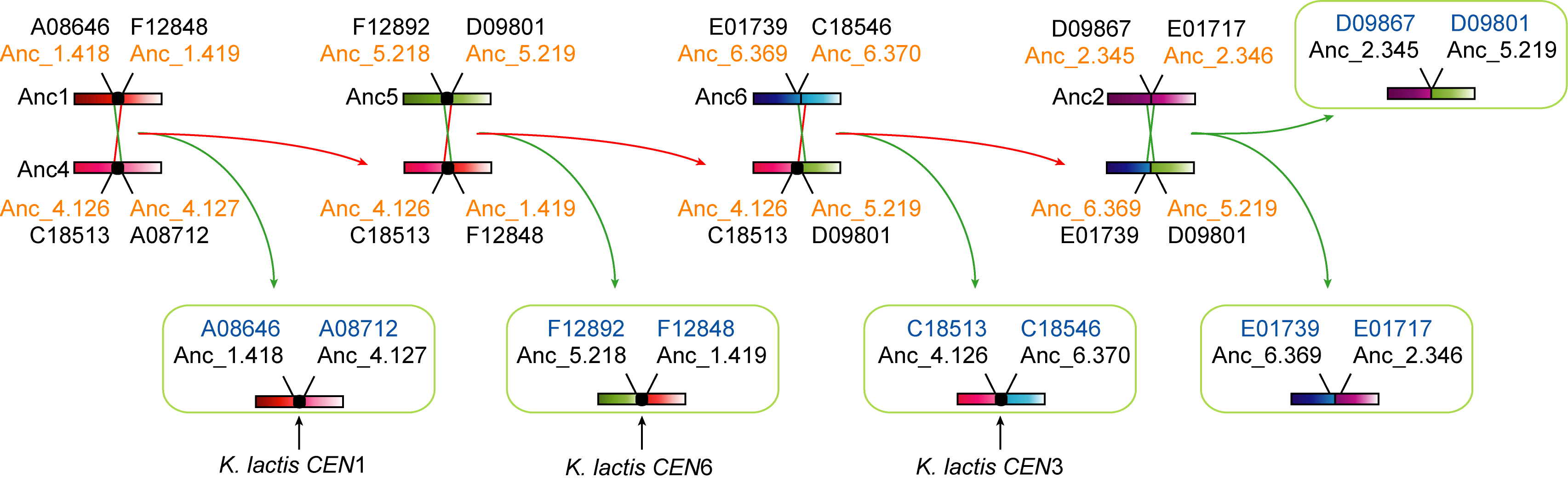

Supplement: Figure S2 — Rearrangement cycle with breakpoint reuse at three centromeric locations in K. lactis. The cycle involves four reciprocal translocation events, three of which occur at Ancestral centromere positions. Each Ancestral centromere-adjacent region is represented by a color gradient block and orange Ancestral gene names. Centromeres are represented by black circles. Reciprocal translocation events are represented by colored lines joining the gradient blocks. Three of the reciprocal translocations produce one ‘finished’ product (indicated by a green arrow, outlined by a green box and with blue K. lactis gene names), which is a current adjacency in the K. lactis genome, and one ‘unfinished’ product (indicated by a red arrow), which will undergo further rearrangement. The final reciprocal translocation produces two ‘finished’ products. (TIF) [file pgen.1002190.s003.tif]

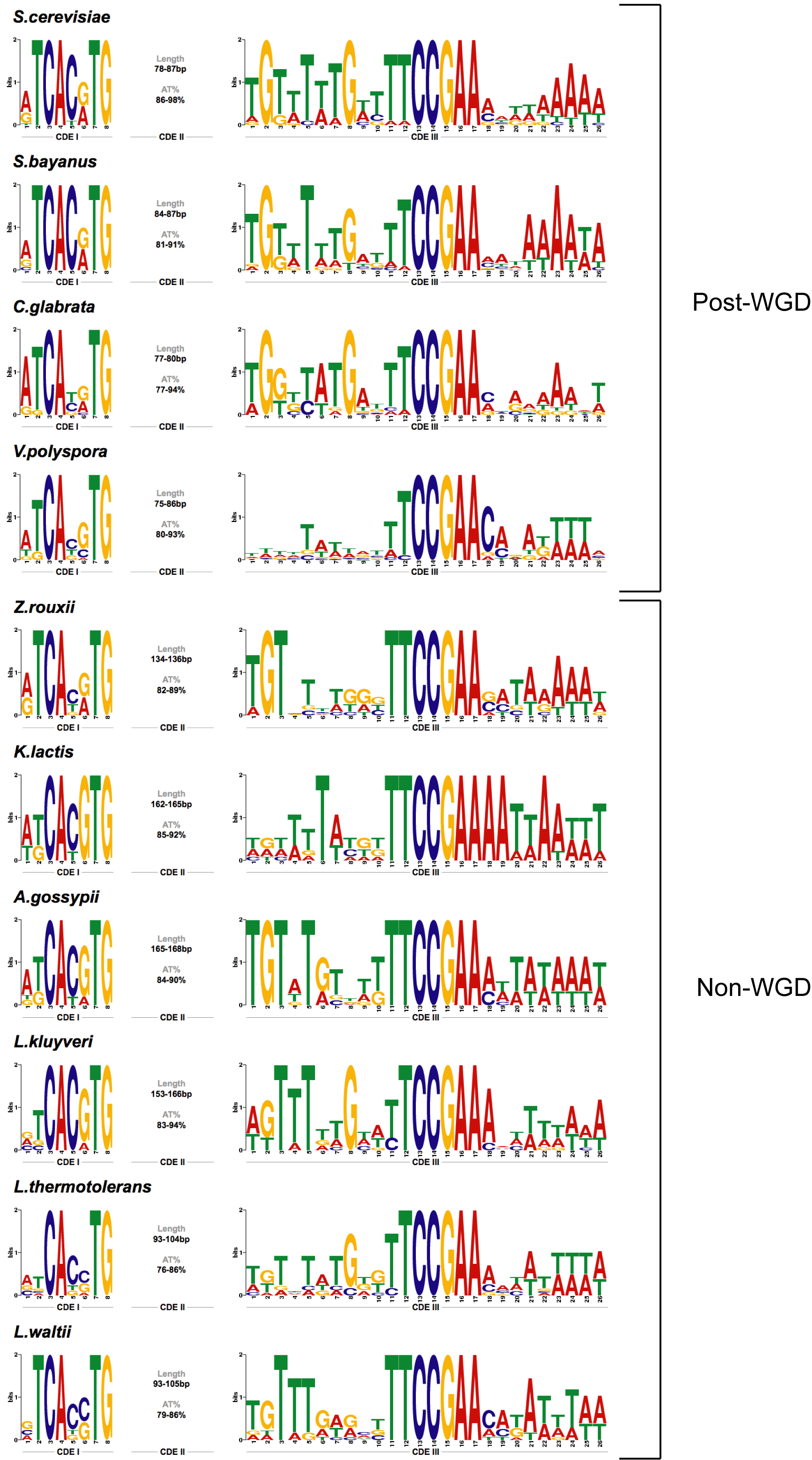

Supplement: Figure S3 — Consensus MEME logos for CDEI and CDEIII motifs in the species examined. Species are split into post-WGD and non-WGD. Length range and %AT range is shown for the CDEII region. (TIF) [file pgen.1002190.s004.tif]
